# Multiple prophage acquisition events over the course of an outbreak drive lysogenic conversion of capsular polysaccharides produced by carbapenem-resistant *Acinetobacter baumannii* isolates

**DOI:** 10.1128/spectrum.04084-25

**Published:** 2026-04-27

**Authors:** Nowshin S. Sharar, Christopher J. Harmer, Ruth M. Hall, Johanna J. Kenyon

**Affiliations:** 1 Centre for Immunology and Infection Control, School of Biomedical Sciences, Faculty of Health, Queensland University of Technology 1969, Brisbane, Australia; 2 School of Life and Environmental Science, The University of Sydney 4334, Sydney, Australia; 3 Sydney Infectious Diseases Institute, The University of Sydney 4334, Sydney, Australia; 4 School of Pharmacy and Medical Sciences, Health Group, Griffith University 5723, Gold Coast, Queensland, Australia; 5 Institute for Biomedicine and Glycomics, Griffith University 5723, Gold Coast, Queensland, Australia; Emory University School of Medicine 12239, Atlanta, Georgia, USA

**Keywords:** *Acinetobacter baumannii*, KL58, capsular polysaccharide, prophage, wzy, polymerase

## Abstract

Prophages are important drivers of bacterial evolution and can provide pathogens with additional factors that enhance virulence and/or resistance properties. However, they are not considered when assessing the dynamics of outbreak populations using phylogenetic approaches. Recently, we showed that BAL114, an extensively antibiotic-resistant *Acinetobacter baumannii* isolate from Vietnam that carries KL58 at the capsular polysaccharide (CPS) biosynthesis locus, produced CPS with two types of linkage between KU58 tetrasaccharide units that were both different from the linkage formed by the Wzy_KL58_ polymerase. Here, we determined the complete genome of BAL114 and identified two *wzy* genes, named *wzy1_Ph_* and *wzy2_Ph_*, in separate prophage that have driven lysogenic conversion by changing the BAL114 CPS. These prophages appear to suppress the action of Wzy_KL58_. Comparison of the BAL114 chromosome with that of BAL062 from the same outbreak sublineage revealed that they shared 99.98% identity over common regions (96% coverage) but differed in prophage content and by recombination-mediated events. Further combinations of prophages with CPS-modifying genes were identified in other members of the same lineage, and a time-calibrated, recombination-free phylogeny revealed multiple prophage acquisitions that could drive structural changes in the CPS had occurred over the short course of the outbreak. The *wzy1_Ph_* and *wzy2_Ph_* genes were detected in several different prophage sequences found in diverse *Acinetobacter* genomes, suggesting that they are being transferred between prophages. This study revealed that prophages may use CPS modification for their own defense and highlighted the importance of considering prophage and recombination when tracing the evolution of bacterial populations.

**IMPORTANCE:**

Capsular polysaccharide (CPS) is a major virulence factor for the critical priority pathogen *Acinetobacter baumannii* and is also the primary receptor for a number of bacteriophages and monoclonal antibodies. Hence, changes in CPS structure may influence virulence potential and substantially impact patient outcomes using novel therapies. Here, we have shown that acquired genes found in prophage can alter the structure of the CPS produced by the chromosomal CPS biosynthesis locus by supplying alternate Wzy polymerases to link the K unit oligosaccharides together. This highlights the need to examine the accessory genome, which we found to be rapidly evolving, as it differed between the two isolates that are closely related based on single-nucleotide polymorphism phylogeny. Other members of the same lineage from the same outbreak carried various prophages that included CPS-modifying genes. Detecting these acquired genes will be needed to underpin the application of alternative therapies.

## INTRODUCTION

Bacteriophage (phage) are natural predators of bacteria that are known to strongly influence the dynamics and evolution of bacterial populations (1, 2). Modulation of bacterial communities can involve virulent phages that participate exclusively in a lytic cycle, leading to cell death, or temperate phages that either enter a lytic cycle or integrate into the bacterial genome to replicate with the host (3). Integrated phage genomes, known as prophages, are generally diverse and often provide resistance to secondary phage infection (1). Prophages can also drive lysogenic conversion via factors they encode that generate phenotypic changes conferring a selective advantage (1). Despite the important implications this may have for enhancing the resistance or virulence of bacterial pathogens, little is known about the extent of lysogenic conversion among members of discrete outbreaks or subpopulations that circulate in a specific clinical or local environment.

Phage adsorption and subsequent infection require the recognition of a receptor(s) on the bacterial cell surface; an interaction that is strongly influenced by the presence of an extracellular polymeric layer surrounding the cells (4, 5). For the critical priority pathogen *Acinetobacter baumannii*, the capsular polysaccharide (CPS) or capsule serves as the initial receptor for phages that encode a tailspike depolymerase (Dpo), allowing them to degrade the CPS (6–8). These Dpo enzymes hydrolyze CPS typically by cleaving the specific glycosidic linkage found between the oligosaccharide K-units (KU) that make up CPS polymers (9, 10). This bond is formed by the activity of a Wzy polymerase, which is usually encoded within the K locus (KL) for CPS biosynthesis in the bacterial chromosome (11, 12). Hence, the host range of each specific phage is largely determined by the structure of the CPS and particularly by the type of Wzy linkage present (7). Phage and their Dpo products are being considered promising therapeutic interventions to treat and control the spread of carbapenem-resistant *A. baumannii* (CRAb), which persists in clinical environments, causing prolonged or recurrent outbreaks of resistant infections (13). Hence, a clear understanding of the factors influencing the structure of the CPS is needed.

**Fig 1:**
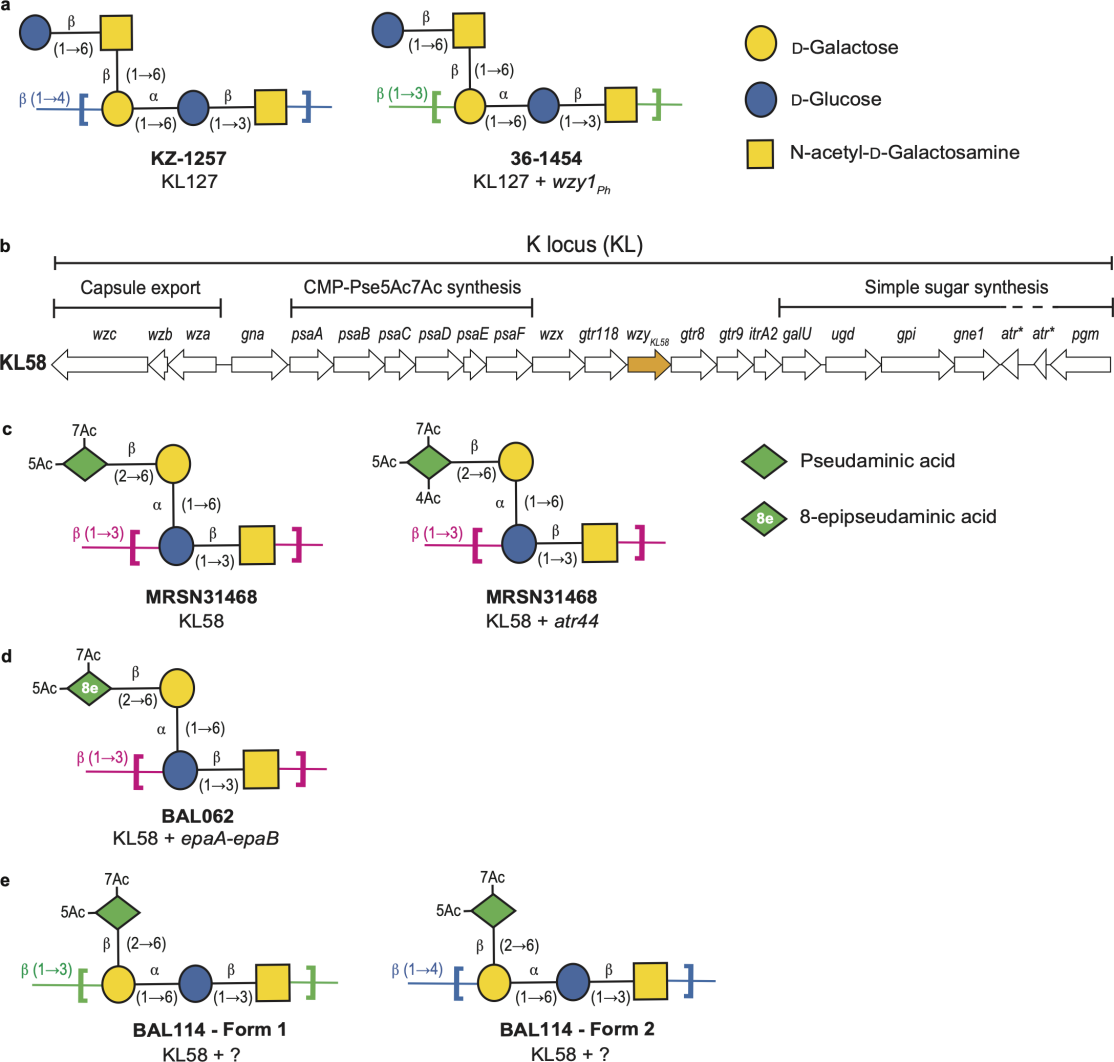
Genetics and structures of capsular polysaccharides produced by *A. baumannii* isolates. (**a**) KZ-1257 CPS and 36-1454 CPS (23). (**b**) Arrangement of the KL58 locus carried by BAL114, BAL062, and MRSN31468. Gene names are shown above, and the *wzy* polymerase gene is colored yellow. (**c**) MRSN 31468 CPS (17). (**d**) BAL062 CPS (15). (**e**) BAL114 CPS (16). Carbohydrates are represented by shapes colored by the symbol nomenclature for glycans (SNFG) scheme (25): glucose (blue circle), galactose (yellow circle), galactosamine (yellow square), and pseudaminic acid isomers (green diamond); 8-epipseudaminic acid is represented by a green diamond with a white 8e inside the diamond. Different linkages between units are indicated by colors. Ac = acetyl.

Several combinations of genes have been found at the chromosomal K locus in *A. baumannii* genomes, and the species has been predicted to produce more than 200 distinct CPS types (12, 14). Generally, where CPS structures have been determined, the structure can be formed using enzymes encoded within the KL (9, 10, 15–21). However, a number of the isolates studied have been found to produce a CPS that requires, or is further modified by, enzymes encoded by genes located in prophages (17, 22–24). For example, it has been shown that the linkage between KU127 pentasaccharides changes (Fig. 1a) when an additional phage-encoded Wzy polymerase, Wzy1_Ph_ (previously called Wzy_Ph1_), is produced in the same cell (23), and the absence of the CPS configuration formed by the KL127-encoded Wzy suggests that the phage is also inhibiting either the production or action of Wzy_KL127_. More recently, two phylogenetically unrelated isolates, named MRSN 31468 and BAL062, both carrying the KL58 CPS biosynthesis locus (Fig. 1b), were found to produce a different tetrasaccharide KU and, hence, a different CPS. The MRSN 31468 KU, KU58 (Fig. 1c), included pseudaminic acid (Pse5Ac7Ac) (17), whereas the BAL062 KU (Fig. 1d) included 8-epipseudaminic acid (8ePse5Ac7Ac), a novel non-2-ulosonic acid isomer (15). The alteration of the Pse5Ac7Ac biosynthesis pathway to produce 8ePse5Ac7Ac is achieved by two enzymes, EpaA and EpaB, which were found to be prophage-encoded (24). In the MRSN 31468 CPS (Fig. 1c), the Pse5Ac7Ac residue was also found to be 4-O-acetylated, and a prophage-encoded acetyltransferase Atr44 was found (17).

In search of another KL58-carrying isolate that produces CPS without prophage modification, we recently solved the CPS structure of a third isolate, named BAL114, which is a close relative of BAL062 but did not carry *atr44* or *epaA-epaB* genes (16). As predicted, this isolate was found to produce CPS made up of KU58 tetrasaccharide units (i.e., 8ePse5Ac7Ac was not present), and the Pse5Ac7Ac was not 4-O-acetylated. However, two different linkages between KU58 units were present (Fig. 1e), both of which were different from the linkage found in the BAL062 and MRSN 31468 CPS that is formed by Wzy_KL58_. Hence, we proposed that two *wzy* genes outside the K locus may produce Wzy polymerases that use KU58 to produce the modified CPS types in BAL114 (16).

BAL062 and BAL114 are CRAb isolates, which were recovered in 2009 during an outbreak of clonal complex 2 (CC2) CRAb that occurred between 2008 and 2012 in the intensive care unit (ICU) at the Hospital for Tropical Diseases (HTD) in Ho Chi Minh City, Vietnam (15, 26). This CRAb outbreak included two distinct phylogenetic lineages, but these two isolates were found to belong to the same sublineage, referred to as sublineage B, of lineage 2. This sublineage is characterized by the presence of a novel Tn*2008VAR* transposon carrying the *oxa23* carbapenem resistance gene (15, 26). Although their phylogenetic relationship is close, prophages, as well as regions of recombination, were excluded from previous phylogenetic studies used to estimate the evolutionary relationships between the outbreak isolates (15, 26). However, our studies on the CPS produced by BAL062 and BAL114 revealed differences and indicate that, at least in the case of conversion of Pse5Ac7Ac to 8ePse5Ac7Ac in BAL062, lysogenic phages have played an important role. This raised the possibility that further prophages have led to the divergence and adaptation of CC2:KL58 isolates over the course of the HTD outbreak and could explain the different Wzy linkages found in the BAL114 CPS.

Here, we have determined the complete genome sequence of *A. baumannii* BAL114 and used it to identify the *wzy* gene candidates responsible for modifying linkages between units in the CPS produced by this isolate. We found two *wzy* genes located in two separate prophages that carry unrelated depolymerases and infer that these prophages were acquired and incorporated into the BAL114 chromosome via independent events that occurred chronologically over the course of the HTD outbreak. We also examine the distribution of the *wzy* genes and the types of prophages that carry them among lineage 2 of the outbreak and more broadly in available *Acinetobacter* genomes.

## RESULTS

### Properties of *A. baumannii* CRAb isolate BAL114

**Fig 2:**
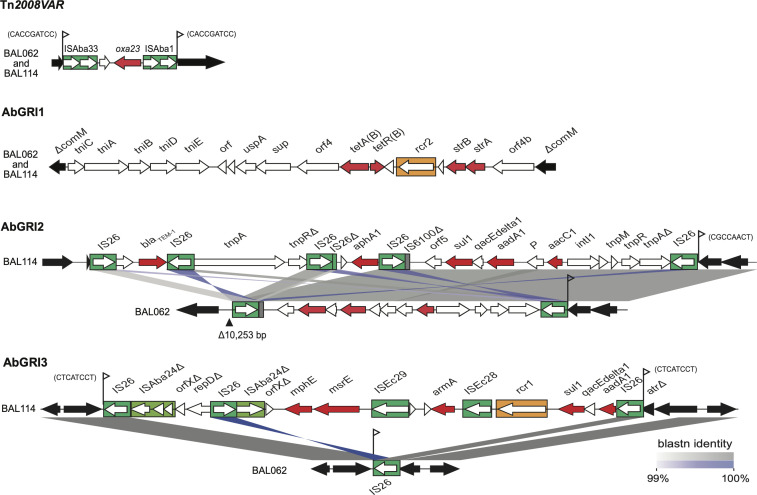
Antibiotic resistance regions identified in the BAL114 chromosome compared with those reported for BAL062 previously (15). The figure was drawn to scale using pyGenomeViz (https://github.com/moshi4/pyGenomeViz) and annotated in Adobe Illustrator. Gray shading and blue shading (inverted matches) for alignments indicate blastn sequence identity as shown by the scale at the bottom right. Antibiotic resistance genes are in red, and IS are indicated by the green boxes.

**Fig 3:**
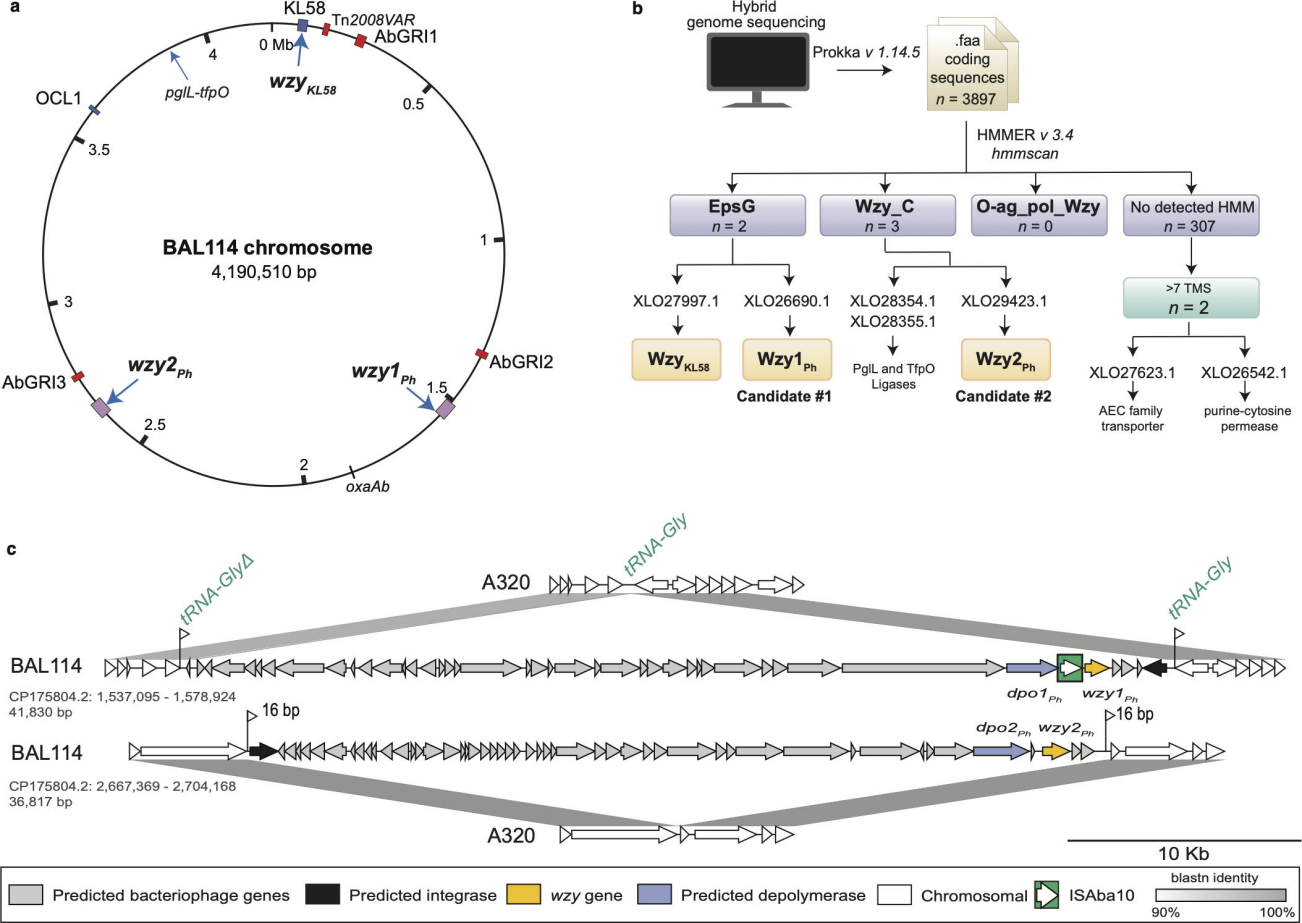
Identification of *wzy* candidates and their genetic context in BAL114. (**a**) Map of BAL114 chromosome showing KL58 and OCL1 loci (blue), and resistance islands (red). Blue arrows inside the circle indicate positions of protein candidates identified via genome screening. (**b**) Workflow used to identify *wzy* candidates. Protein accession numbers for candidates identified in the BAL114 chromosome are shown. (**c**) Comparison of prophage regions carrying *wzy1_Ph_* and *wzy2_Ph_* genes, indicating their boundaries and position of insertion in the A320 reference chromosome. Flags indicate sequence duplication at the target site. Alignments generated using pyGenomeViz (https://github.com/moshi4/pyGenomeViz) and annotated in Adobe Illustrator.

A complete genome was obtained for *A. baumannii* BAL114 via sequencing using Oxford nanopore and Illumina technologies. The hybrid assembly included a 4,190,510 bp chromosome (NCBI accession number CP175804.1) and an 11,216 bp r3-T4 plasmid (NCBI accession number CP175805.1). BAL114 belongs to ST2 of CC2 and carries the KL58 and OCL1 loci as described previously (26). BAL114 was extensively resistant. It was found to be resistant to ampicillin, ceftazidime, cefotaxime, cefepime, carbapenems, imipenem, meropenem, and doripenem. It was also resistant to all aminoglycosides used clinically (gentamicin, tobramycin, and amikacin) and to further aminoglycosides tested (streptomycin and spectinomycin, kanamycin and neomycin, and netilmicin), as well as sulfonamides, trimethoprim. BAL114 was resistant to tetracycline and doxycycline but susceptible to minocycline; however, it was also resistant to fluoroquinolones, ciprofloxacin, and levofloxacin. Consistent with the carbapenem-resistant phenotype, BAL114 carried the *oxa23* gene in the transposon Tn*2008VAR* (Fig. 2), which has, to date, only been found in the chromosome (see Fig. 3a for location) of a subgroup of CC2 isolates from Vietnam, including BAL062 (15, 26). Cephalosporin resistance is accounted for by the presence of an appropriately oriented ISAba1 located 9 bp upstream of the intrinsic chromosomal *ampC* gene (27, 28), and fluoroquinolone resistance is due to mutations in *gyrA* and *parC* that introduce an S81L substitution in GyrA and an S84L substitution in ParC.

BAL114 carries multiple acquired resistance genes in variant forms of the CC2-associated genomic resistance islands (GRIs) AbGRI1, AbGRI2, and AbGRI3 (29, 30) located in the chromosome at positions indicated in Fig. 3a, and these genes account for the remaining resistance phenotypes. The structures of these GRI are compared to those in its close relative BAL062 (NCBI accession number LT594095.1 [15]) in Fig. 2. While AbGRI1 in BAL114 is identical to the form found in BAL062, both the *bla*_TEM_ and *aphA1* genes present in AbGRI2 in BAL114 have been lost from BAL062, together with a 10 kbp segment of the chromosome adjacent to the left end of AbGRI2 in BAL114. In addition, in BAL114, a variant form of AbGRI3 (see [30] for complete AbGRI3 structure) includes the *armA* gene (resistance to all used aminoglycosides), whereas only the left-hand bounding IS*26* remains in BAL062 as the complete resistance region plus a small segment of the chromosome on the right has been deleted. These events have likely occurred as a result of the action of IS*26* (31).

### *A. baumannii* BAL114 carries two additional Wzy polymerase genes

To identify candidate genes coding for Wzy polymerases in the BAL114 genome, all coding sequences (*n* = 3,897) were assessed for features consistent with Wzy sequences as outlined in the workflow shown in Fig. 3b. This process identified several sequences with >7 transmembrane segments (TMS) that belong to the EpsG (*n* = 2; Interpro accession number IPR049458) and Wzy_C (*n* = 3; Interpro accession number IPR007016) families that are associated with Wzy polymerases (10). One of the EpsG family proteins was identified as Wzy_KL58_ (326 amino acids; GenPept accession number XLO27997.1) encoded at the K locus, and two of the Wzy_C proteins were identified as the known PglL (GenPept accession number XLO28355.1) and TfpO (GenPept accession number XLO28354.1) ligases required for protein and pilin glycosylation in *Acinetobacter,* respectively (32). These sequences were excluded from further analysis, leaving two candidates remaining (Fig. 3b).

The first candidate (347 amino acids; GenPept accession number XLO26690.1) belongs to the EpsG family and was found to share 99.14% aa identity (100% coverage) with the prophage-encoded Wzy1_Ph_ polymerase (GenPept accession number MBV6766733.1) that has been shown to be responsible for forming β-d-Gal*p*NAc-(1→3)-d-Gal*p* linkages between KU127 pentasaccharide units (Fig. 1a) (23). As a β-d-Gal*p*NAc-(1→3)-d-Gal*p* linkage is also found between KU58 tetrasaccharide units in one of the CPS forms in BAL114 (Fig. 1e), this coding sequence was assigned to formation of this linkage.

The second candidate (392 amino acids; GenPept accession number XLO29423.1) belongs to the Wzy_C family. Although it did not share any significant identity (>25% aa identity over >75% coverage) with any Wzy previously identified in *A. baumannii* (12), the sequence was found to be 27.3% aa identical (75% coverage) to a Wzy (GenPept accession number AXZ00116.1) encoded by the *Proteus mirabilis* O28 O-antigen gene cluster. The *P. mirabilis* O28 O-antigen includes a β-d-Glc*p*NAc-(1→4)-d-Gal*p*A linkage between oligosaccharide units (33), which is similar to the β-d-Gal*p*NAc-(1→4)-d-Gal*p* linkage in the second BAL114 CPS form (Fig. 1e). Granted that the second candidate shared similarity with a known polymerase and that the linkages between units are similar, the product was considered a good candidate for the formation of the β-d-Gal*p*NAc-(1→4)-d-Gal*p* linkage in the BAL114 CPS. It was named Wzy2_Ph_ for reasons described below.

### The additional Wzy polymerases are encoded in prophage

**Fig 4:**
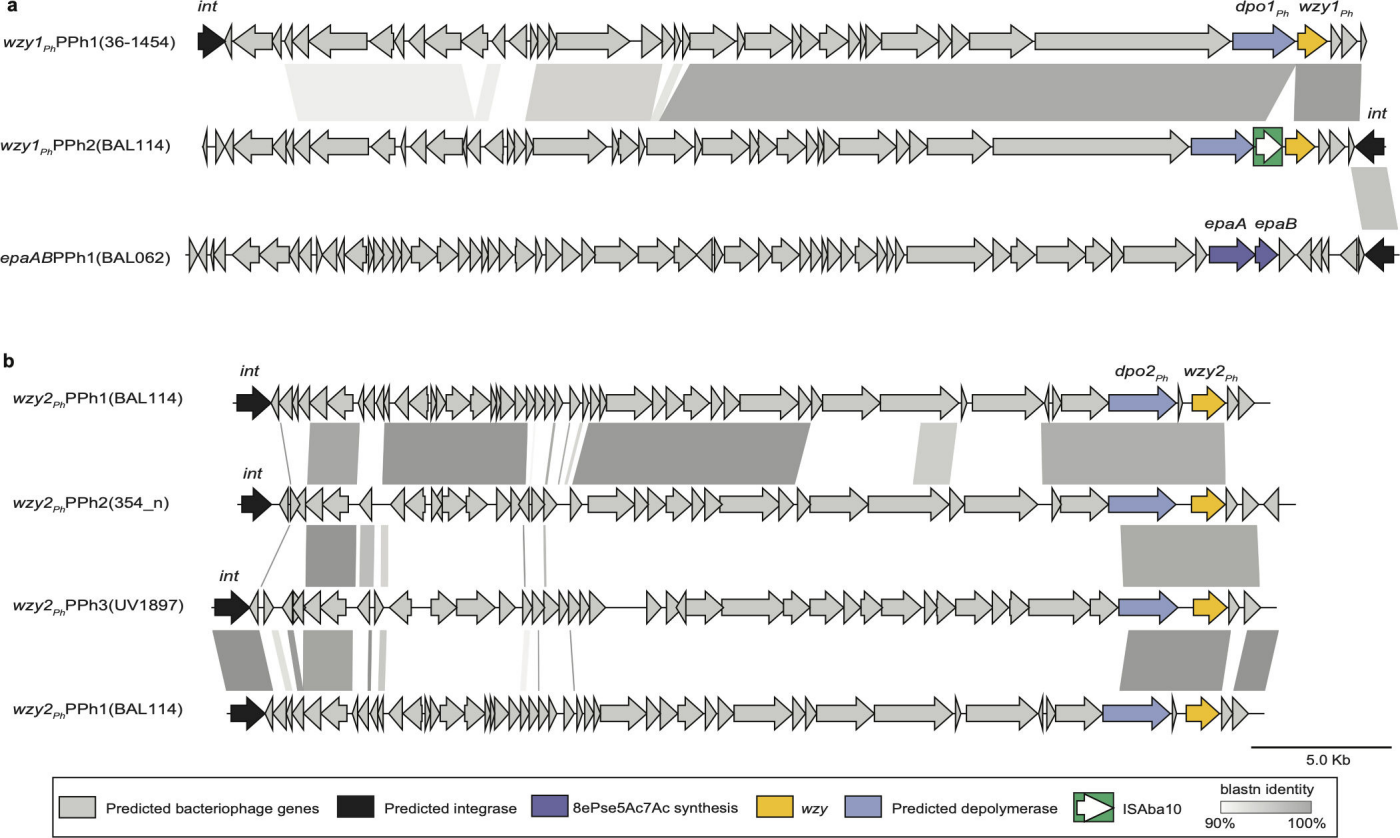
Prophage comparisons. (**a**) Alignment of the prophages from BAL114 carrying *wzy1_Ph_* with the prophages from 36-1454 (23) and prophages with *epaA-epaB* from BAL062 (24). (**b**) Comparison of prophages with *wzy2_Ph_* identified in genomes of HTD isolates. Color scheme and scales are shown at the bottom of the figure. Alignments were generated using pyGenomeViz (https://github.com/moshi4/pyGenomeViz) and annotated in Adobe Illustrator.

In the BAL114 chromosome, the coding sequence of the *wzy1_Ph_* gene was found close to genes associated with bacteriophage formation, including one predicting a depolymerase designated here as Dpo1_Ph_ (NCBI accession number XLO26692.1). To identify the boundaries of this region, the gene and the surrounding sequence were aligned with the A320 CC2 reference genome (NCBI accession number CP032055.1 [34]). This revealed a 41.8 kbp insertion (CP175804.2: bases 1,537,095–1,578,924) in a tRNA-Gly gene. This insertion corresponds to the genome of a prophage belonging to the Class *Caudoviricetes* that includes a partial tRNA-Gly sequence (Fig. 3c). It includes 41 predicted open reading frames (ORFs), among which one gene located at one end encodes a tyrosine recombinase/integrase that would catalyze prophage integration. The *dpo1_Ph_* and the *wzy1_Ph_* genes are located nearby but are separated by an ISAba10 insertion sequence. This prophage, hereafter referred to as *wzy1_Ph_*-PPh2 (PPh = Prophage), shares 97.23% DNA sequence identity (84% coverage) with the previously identified prophages that include *wzy1_Ph_* in isolate 36-1454 (here designated *wzy1_Ph_*-PPh1; Fig. 4a).

The coding sequence for the second candidate, Wzy2_Ph_, was similarly located close to genes associated with phage. Alignment of the surrounding region in the BAL114 chromosome with the A320 genome revealed a 36.8 kbp insertion (Fig. 3c; CP175804.2: bases 2,667,369–2,704,168) and two copies of a 16 bp segment (one from the prophage and one from the chromosome) that includes the end of a gene annotated as encoding a TamB translocation and assembly module protein. This region was also found to consist of a complete prophage belonging to the Class *Caudoviricetes*. This prophage, designated *wzy2_Ph_*-PPh1, includes 48 predicted ORFs, including an *int* gene encoding a tyrosine recombinase/integrase at one end and a depolymerase *dpo2_Ph_* and the *wzy2_Ph_* gene near the other end. It shared no sequence identity with *wzy1_Ph_*-PPh2 or *wzy1_Ph_*-PPh1. The locations of the two prophages are indicated in Fig. 3a.

### Has *wzy1_Ph_*-PPh2 prophage displaced the *epaAB*-PPh1 prophage in BAL062?

We noticed that the *wzy1_Ph_*-PPh2 prophage in BAL114 is in the same location as the *epaAB-*PPh1 prophage in BAL062 and that both had been incorporated into a tRNA-Gly gene. Consistent with this, the encoded integrases are 99.7% identical (Fig. 4a). However, while the likely end of the Wzy1_Ph_ prophage in BAL114 had a clear junction with the A320 chromosomal sequence at the *int* end, at the other end, it was flanked by a diverged region, returning after approximately 330 bp to a high level of identity (>99%) to A320. Comparison of this region in the complete chromosomes of BAL114, BAL062 (GenBank accession number LT594095.1), and A320 revealed that on that side, the BAL062 sequence was identical to A320. The diverged segment extended for about 28.9 kbp, and searches revealed that it was >99% identical to the corresponding segment of the chromosome in CC10 isolates, consistent with it being an imported recombinant region. This configuration is consistent with the acquisition of the *wzy1_Ph_*-PPh2 prophage via uptake of the region containing it from an *A. baumannii* CC10 isolate, followed by the incorporation of a large segment via site-specific incorporation at one end (the *int* end of the prophage) and homologous recombination at the other. With this scenario, it is not possible to distinguish the replacement of the *epaAB* prophage from incorporation into an uninterrupted tRNA-Gly.

### Role of prophage in the divergent evolution of isolates BAL114 and BAL062

**Fig 5:**
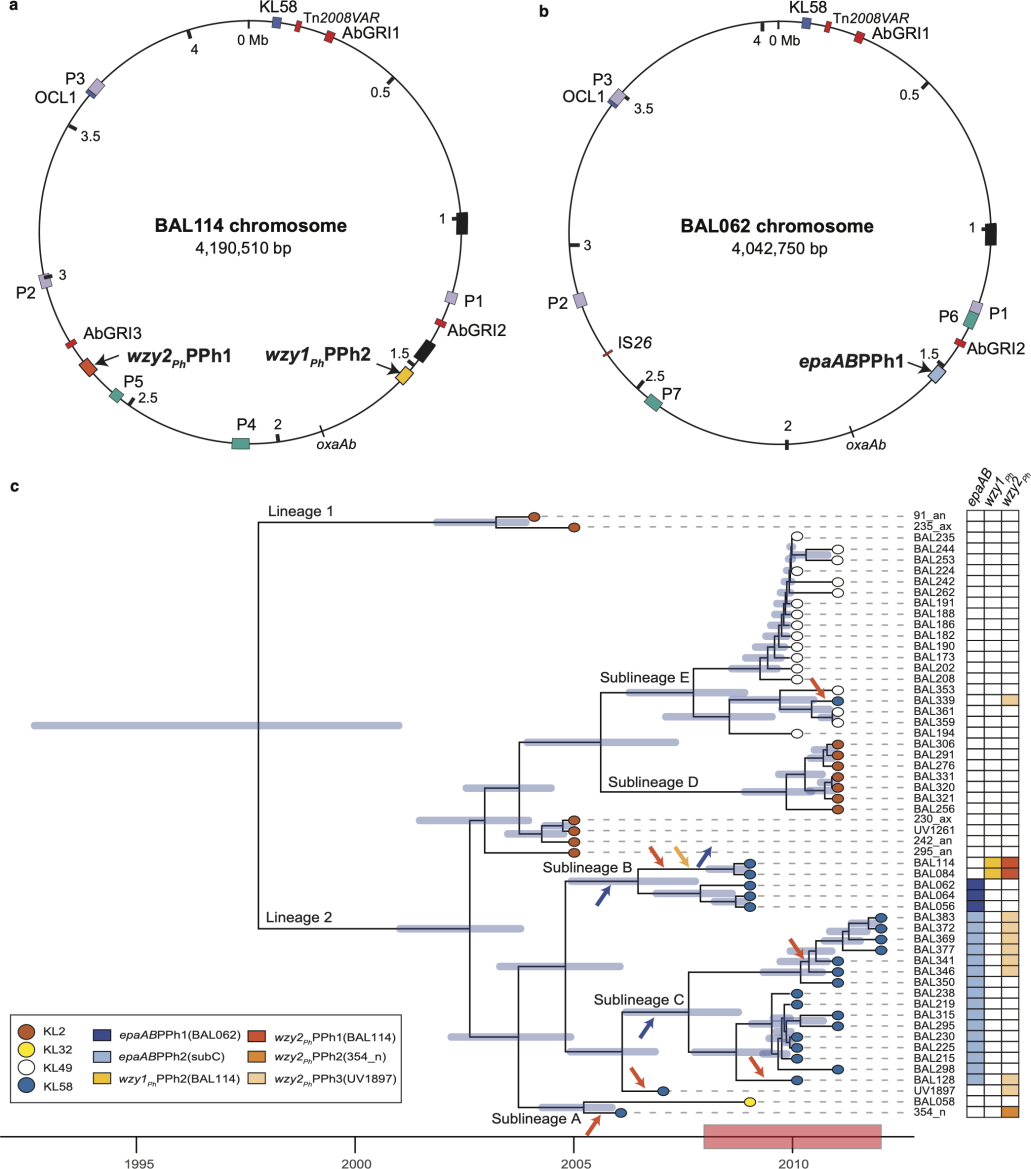
Acquisition of prophage in isolates recovered from the HTD. (**a**) Map of BAL114 chromosome compared with (**b**) the map of the BAL062 chromosome. Sequences have been reverse-complemented to reflect the same chromosomal orientation used routinely. Features shared are indicated as labels outside circles, and differences are indicated as labels inside circles. Shared prophages are in purple, *epaAB-*PPh is in blue, *wzy1_Ph-_*PPh2 is in yellow, and *wzy2_Ph-_*PPh1 is in orange. Other prophages that are different are in green. Black boxes are the positions of ~80 kbp duplicated sequence. (**c**) Time-calibrated core-single-nucleotide polymorphisms (SNP) phylogeny of CC2 isolates recovered at the HTD overlaid with information relating to KL and prophage regions. Blue horizontal bars indicate 95% CI calculated by BactDating. Years of ICU outbreak are indicated by a red box shown on the time scale below. Nodes are colored by KL with the key shown at the bottom left. Prophages are indicated in the panels to the right. Arrows along the branches indicate prophage acquisition or displacement and are colored according to the key.

The chromosome sequences of BAL114 (Fig. 5a) and BAL062 (Fig. 5b), which are both members of CC2:L2 sublineage B, were compared in order to identify any additional regions of difference between the two branches of sublineage B. The chromosomes were found to share 99.98% DNA identity over common regions (96% chromosome coverage) that included KL58, OCL1, Tn*2008VAR,* and the AbGRI1 resistance island (Fig. S1). Although they differ in the regions outside prophage genomes by 455 SNDs, 411 are in the 28.9 kbp patch described above. The remaining 44 SNDs are distributed throughout the chromosome. BAL062 and BAL114 include copies of the insertion sequence ISAba1 at the same 12 positions along the length of the chromosome, but BAL114 includes an additional four ISAba1 copies. These features are consistent with a recently shared history as revealed in the time-dated phylogeny (Fig. 5c). However, other events have led to diversification.

Predicted prophages in both BAL114 and BAL062 were identified using PHASTEST (Table 1) and their locations mapped onto the relevant chromosome (Fig. 5a and b). For simplicity, additional prophages are labeled as P1–P7 in Fig. 5. Alignment of the two chromosomes (Fig. S1) revealed that only three prophages (P1–P3) were present in both isolates. P4, P5, *wzy1_Ph_*-PPh2, and *wzy2_Ph_*-PPh1 are present in BAL114 (Fig. 5a), and prophages P6, P7, and *epaAB*-PPh1 are in BAL062 (Fig. 5b). P6 had been incorporated adjacent to P1, and similar to *wzy1_Ph_*-PPh2 and *epaAB*-PPh1, P5 in BAL114 was found to be in the same position as P7 in BAL062. Hence, multiple prophage acquisition events had occurred in each branch of sublineage B. The alignment shown in Fig. S1 also revealed that BAL114 included a second copy of an ~80 kbp region that is located ~0.4 Mb away (Fig. 5a; black boxes). However, although the repeated regions were closely related, they were not precise matches, and clear boundaries were not identified. Both regions encode several ORFs associated with bacteriophage and may represent prophage or remnant(s) of prophage not detected by PHASTEST.

**TABLE 1:** Prophage identified in *A. baumannii* BAL114 and BAL062 chromosomes

Prophage region	Length (Completeness)	CPS genes	Chromosome location^*d*^	
			BAL114	BAL062
*wzy1*_*Ph*_PPh2	41.8 kbp (Intact^*b*^)	*wzy1*_*Ph*_	1,537,095–1,578,924	–
*wzy2*_*Ph*_PPh1	36.8 kbp (Intact^*b*^)	*wzy2*_*Ph*_	2,667,369–2,704,168	–
*epaAB*PPh	43.1 kbp (Intact^*b*^)	*epaA-epaB*	–	1,454,437–1,497,501
P1	35.1 kbp (Intact^*a*^)		1,246,351–1,281,498	1,227,769–1,262,916
P2	43.1 kbp (Intact^*a*^)		2,964,907–3,008,056	2,799,624–2,842,773
P3	33.2 kbp (Intact^*a*^)		3,627,937–3,661,180	3,461,480–3,494,723
P4	42.8 kbp (Intact^*c*^)		2,112,265–2,155,120	–
P5	34.2 kbp (questionable^*a*^)		2,545,385–2,579,642	–
P6	50.3 kbp (Intact^*a*^)		–	1,254,915–1,305,311
P7	52.6 kbp (Intact^*a*^)		–	2,404,663–2,457,321

^*a*^ Determined using PHASTEST.

^*b*^ Determined by manual inspection in this and a previous study (24).

^*c*^ Determined using PHASTER.

^*d*^ “–” indicates that prophage is not present.

### Occurrence of different prophages carrying CPS biosynthesis genes among HTD isolates

The short-read data previously reported for a further 62 CC2 isolates from the HTD (26), including those recovered from the ICU outbreak (35) as well as asymptomatic carriage and infection isolates recovered from the ICU in the years prior (36), were screened for *wzy1_Ph_* and *wzy2_Ph_* genes. Only BAL114 and BAL084 were found to carry *wzy1_Ph_* (Table S1). However, the *wzy2_Ph_* gene was detected in 10 additional KL58-carrying isolates (Fig. 5c and Table S2), all of which are known members of lineage 2 (15, 26). The earliest, 354_n, a nasal carriage isolate recovered in 2006 prior to the start of the outbreak, had *wzy2_Ph_* in a different prophage, designated *wzy2*_Ph_-PPh2(354_n). The nine other isolates carried *wzy2_Ph_* in a third prophage, named *wzy2*_Ph_-PPh3(UV_1897). The earliest of these, UV_1897, was recovered in 2007 from tracheal wash, whereas the remaining eight isolates were recovered between 2010 and 2012 from bronchoalveolar lavage (BAL) samples taken from patients with ventilator-associated pneumonia (26). This genomic data indicated that *wzy2_Ph_* was circulating in isolates prior to the start of the outbreak.

To assess genetic relatedness, the prophages were compared with each other (Fig. 4b). *wzy2*_Ph_-PPh2(354_n) was found to share 99.05% sequence identity over 81% of its 37.3 kbp length with *wzy2*_Ph_-PPh1(BAL114). However, *wzy2*_Ph_-PPh3(UV_1897) was quite different. Although regions of similarity between *wzy2*_Ph_-PPh3(UV_1897) and *wzy2*_Ph_-PPh1(BAL114) or *wzy2*_Ph_-PPh2(354_n) represented less than 25% sequence coverage (Fig. 4b), the sequence of the *wzy2_Ph_* gene was identical in all three prophages. The sequence identity between *wzy2*_Ph_-PPh3(UV_1897) and *wzy2_Ph_*-PPh2(354_n) extends further upstream into the coding region for the C-terminal domain of the predicted depolymerase and for the two ORFs immediately downstream, suggesting that this portion of sequence may have been recently transferred between them. The locations of these prophages in the HTD genomes were also traced as described above. *wzy2*_Ph_-PPh3(UV_1897) was precisely inserted into the *tamB* gene of A320 (locus tag A320_01452) in all genomes that carried this prophage, and this is the position of *wzy2*_Ph_-PPh1(BAL114) in BAL114. However, the location of *wzy2_Ph_*-PPh2(354_n) was different. It was inserted close to the start of a *gspL* gene that codes for a type II secretion system protein (NCBI accession number XLO29251.1), located ~60 kbp away from AbGRI3.

Among the ST2 isolates recovered from the HTD, prophages carrying *wzy1,_Ph,_ wzy2_Ph_*, or *epaA-epaB* were only found in those that carried the KL58 locus. Previously, it was shown that the KL58 locus was likely acquired into an ancestral ST2 isolate via a recombination event involving a CC10 isolate (26). Hence, all short-read data for non-CC2 isolates (*n* = 81) previously reported from the HTD (26) were also screened for the presence of *wzy1_Ph_* and *wzy2_Ph_*. No further isolates were found to include *wzy1_Ph_*. However, *wzy2_Ph_* was found in five CC10 (ST575) isolates that also carry KL58. All of these isolates carried *wzy2_Ph_*-PPh2(354_n) (Table S2), and like 354_n, were carriage isolates that had been recovered prior to the start of the outbreak in 2005 (*n* = 1) or 2006 (*n* = 4).

### Evidence of multiple prophage acquisitions prior to and throughout the HTD ICU outbreak

To better understand the temporal dynamics and acquisition of prophages carrying CPS biosynthesis genes among members of the population, the time-calibrated phylogenetic tree of HTD ST2 isolates previously reported in a previous study (26) was reconstructed using all ST2 lineage 2 isolates, as well as two ST2 lineage 1 isolates as an outgroup (Fig. 5c). This recombination-filtered core-SNP phylogeny was overlaid with information relating to KL and prophages carrying either *wzy1_Ph_, wzy2_Ph_*, or *epaA-epaB* (Fig. 5c). *wzy1_Ph_*-PPh2(BAL114) and *epaAB*-PPh1(BAL062) were restricted to sublineage B, while the other prophages with *epaAB* (here named *epaAB*PPh3-(subC)) were found in all members of sublineage C as reported previously (24). However, prophages with *wzy2_Ph_* were distributed more broadly across lineage 2, found in isolates belonging to sublineage A (*wzy2_Ph_*-PPh2(354_n)), B (*wzy2_Ph_*-PPh1(BAL114)), C (*wzy2_Ph_*-PPh3(UV_1897)), or E (*wzy2_Ph_*-PPh3(UV_1897)). Prophage *wzy2_Ph_*-PPh3(UV_1897) was the only sequence identified across multiple sublineages, and the finding of this region in two separate clades of sublineage C indicates that this prophage was likely acquired on more than one occasion following acquisition of *epaAB*PPh3-(subC).

### Distribution of *wzy1_Ph_* and *wzy2_Ph_* in *A. baumannii* genomes

Publicly available *Acinetobacter* genome sequences in the NCBI non-redundant and WGS databases (15 May 2025) were screened for the presence of *wzy1_Ph_* and *wzy2_Ph_* genes to assess the distribution of these genes and their genetic context more broadly. Besides BAL114 and BAL084, no other genome was found to carry both *wzy1_Ph_* and *wzy2_Ph_*. The *wzy1_Ph_* gene was only identified in a further 15 genome sequences (Table S1), which, together with BAL114, BAL084, and 36-1454, represent a total of 18 isolates. The *wzy2_Ph_* gene was detected in an additional 25 genome sequences (Table S2), which, together with BAL114 and the HTD isolates, represented a collective total of 42 isolates.

**Fig 6:**
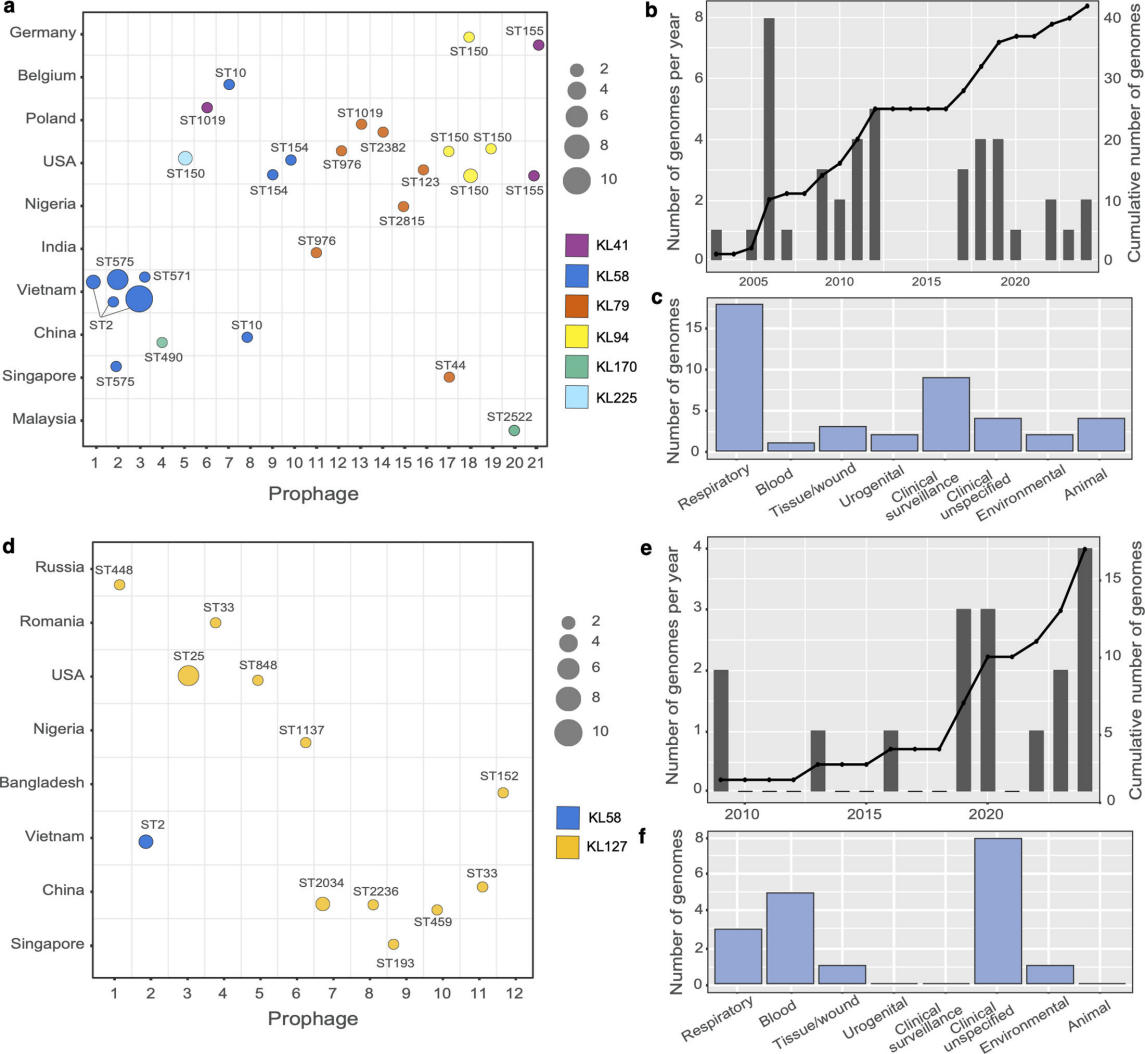
Distribution of *wzy2_Ph_* (**a, b, c**) and *wzy1_Ph_* (**d, e, f**) in *A. baumannii* genomes.

The 42 genomes carrying *wzy2_Ph_* were found to belong to 15 different STs (Table S2). They were distributed across 10 countries (Fig. 6a) and were recovered between 2003 and 2024 (Fig. 6b) largely from clinical sources (Fig. 6c). A total of 21 distinct prophage regions carrying *wzy2_Ph_* were detected (Fig. S2), and again, these were all found to belong to the class *Caudoviricetes* (Table S3). Most included a gene for a predicted depolymerase. However, nearly half had low completeness scores, suggesting that most of these sequences were not intact or were incomplete due to contig breaks in draft genomes. One isolate, named NCGM 193, which was found to carry KL58 with *wzy2_Ph_*-PPh3(UV_1897), had been recovered in 2011 at the Cho Ray Hospital in Ho Chi Minh City, indicating a possible transfer event to a nearby hospital. Another isolate, SIMBA005 (NCBI accession number GCA_047301355.1) recovered in Singapore in 2006, was found to carry *wzy2_Ph_*-PPh2(354_n) with KL58. The remaining prophages were found in genomes carrying either KL41, KL79, KL94, KL170, or KL225, and structural data on the CPS are not available for these KL. In other *Acinetobacter* species, *wzy2_Ph_* was found in *Acinetobacter pittii* (*n* = 3)*, Acinetobacter oleivorans* (*n* = 1), *Acinetobacter geminorum* (*n* = 1), and *Acinetobacter* sp. (*n* = 1) (Table S2).

The 18 isolates carrying *wzy1_Ph_* belonged to 12 different STs and were recovered from eight countries (Fig. 6d) between 2009 and 2024 (Fig. 6e). The majority (*n* = 17) were also from clinical sources (Fig. 6f). All isolates, except for BAL114 and BAL084, carried KL127. In all genomes, *wzy1_Ph_* was found in a prophage of the class *Caudoviricetes* with a total of 12 different sequences identified (Fig. S3, Table S4). Many sequences had high completeness scores with identifiable integrase genes at one of the termini, suggesting that the prophages were intact. However, two appeared to be remnant or incomplete sequences. A putative depolymerase domain (IPR012334) could be identified in the C-terminus of the tail spike protein encoded by all but one prophage, with this protein domain sharing significant identity (>80%) consistent with these enzymes having specificity for the same CPS linkage.

## DISCUSSION

While it is widely appreciated that lytic and lysogenic phages play an important role in shaping bacterial communities, the prophage content of bacterial genomes and the effects of these prophages are rarely addressed. Here, we have identified two prophages in the BAL114 chromosome that encode CPS-modifying enzymes that can account for the differences observed in the BAL114 CPS relative to that of MRSN 31468, which makes K58 CPS (17). Because the specificity of Wzy1_Ph_ was known (23), it was possible to assign Wzy2_Ph_ to the second type of linkage in BAL114 CPS. However, determination of the structure of CPS made by a strain carrying only KL58 and *wzy2_Ph_* would confirm this assignment. Potential alternate treatment strategies for extensively antibiotic-resistant *Acinetobacter* infections, such as antibody therapy and phage therapy, are impacted by the specific nature of the CPS. This is clearly demonstrated in a recent study that showed that 4-O acetylation of Pse5Ac7Ac rendered the CPS produced by MRSN 31468 undetectable using an antibody generated to detect Pse (37). To facilitate future studies on the broader distribution of *wzy* genes in *A. baumannii*, the *Kaptive* database that is currently widely used for *in silico* prediction of CPS type (12, 14) has been updated to include *wzy2_Ph_*. However, it is possible that further distinct *wzy* genes in prophages may lie undetected in *A. baumannii* genomes, and strategies to detect and track these will be needed.

How the activity of the Wzy polymerases encoded by the K locus in both BAL114 (KL58) and 36-1454 (KL127) is suppressed needs to be further investigated. However, it seems likely that the prophage in 36-1454 (KL127) and one of the prophages in BAL114 are responsible for this effect. One option is the production of a specific Wzy inhibitor, as has been reported in a case in *Pseudomonas aeruginosa* (38). For BAL114, it remains unclear why entry of the second prophage does not inhibit Wzy2_Ph_ produced by the prophage with the earlier occupancy, but the presence of ISAba10 upstream of the *wzy1_Ph_* gene in Wzy1_Ph_PPh2 could be a factor. In the case of prophage that, like the ones described here, include genes for both a tailspike depolymerase and a Wzy, it can be speculated that after entry into a suitable host using the Dpo, inhibition of the Wzy_KL_ and production of an alternative CPS configuration could protect the cell carrying the prophages from attack by close relatives if the Dpo does not recognize the new CPS form. Further studies on Dpo specificity will be needed to establish if this is so. However, as the Wzy1_Ph_PPh2 sequence may not have been acquired as a result of an encounter with a phage, the activity or specificity of Dpo1_Ph_ cannot be assumed.

More broadly among members of the same lineage of the HTD outbreak, mapping the prophages that encode CPS-modifying enzymes onto a phylogenetic tree (Fig. 5) provided evidence that multiple prophage acquisition events occurred over the course of the HTD outbreak. It also revealed that *wzy2_Ph_*-PPh3(UV_1897) was acquired into sublineage C on more than one occasion and had been further transferred into sublineage E, which is consistent with multiple events rather than a single acquisition event followed by clonal expansion. The analysis further revealed two isolates that carry the *wzy2_Ph_* gene without the *epaA-epaB* genes and several sublineage C isolates with both *epaAB* and *wzy2*_Ph_. These isolates would be predicted to produce further variant CPS forms using either KU58 or the KU58e (with 8ePse replacing Pse). With *wzy2*_Ph_, these isolates would be predicted to produce CPS that include β-d-Gal*p*NAc-(1→4)-d-Gal*p* linkages between KU. Given that even minor changes to the carbohydrate composition of K-units that comprise CPS polymers have been shown to significantly enhance virulence via preventing adhesion and phagocytosis, and reducing bacterial clearance *in vivo* (39), it is possible that these prophages could confer fitness advantages/costs for lineage 2 isolates. Some comparative studies have been undertaken on isolates of the HTD outbreak (40, 41), but the virulence properties of BAL114 have not yet been examined or compared with other lineage 2 members.

In phylogenomic analyses assessing the dynamics and evolution of outbreak or clonal isolates, factors other than SNPs are not often considered. However, recently, more additional features (resistance genes present, KL, OCL) are being mapped onto trees (26, 42–47). Indeed, the need to include genomic structural variation into account has been highlighted recently (48), and this is facilitated by genomes determined using long reads to assemble a complete genome. In this study, we found extensive short-term diversification in two closely related isolates belonging to the same discrete sublineage of an outbreak via comparison of complete genome sequences. BAL114 and BAL062 diversified predominantly via the incorporation of different prophage but also via IS*26*-mediated changes to two of the antibiotic resistance islands, acquisition of additional ISAba1 insertions, and the recombination-mediated incorporation of a divergent sequence replacing an original region. The presence of polysaccharide biosynthesis genes in prophage resident in bacterial genomes has been reported previously, not only in *A. baumannii* (17, 22–24) but also in several other bacterial species (49), including a report of prophages carrying an entire CPS biosynthesis locus (50). This highlights the need to also account for prophage when assessing structural differences to trace the evolution of bacterial populations.

## MATERIALS AND METHODS

### Bacterial isolates, cultivation, and antibiotic resistance profiling

*A. baumannii* isolate BAL114 is a CC2:ST2:KL58 carbapenem-resistant isolate that had been reported previously (26). The isolate had been recovered in 2009 from a patient suffering from ventilator-associated pneumonia in the HTD intensive care unit. The isolate was grown at 37°C in Luria-Bertani medium. Resistance to antibiotics was determined as described previously (42).

### Whole genome sequencing and identification of Wzy candidates

Whole cell DNA was recovered from a single colony of BAL114 by column purification, and the concentration and purity were measured using a Qubit fluorometric quantification assay. Purified high-quality DNA was sent for hybrid sequencing (Illumina and Oxford Nanopore Technology [ONT]) at Plasmidsaurus, USA. Read quality was checked using FastQC (https://qubeshub.org/resources/fastqc), and ONT reads were filtered to remove reads <1,000 bp using Filtlong *v* 0.2.1 (https://github.com/rrwick/Filtlong). The sequence was assembled using Unicycler *v* 0.5.0 with default settings (51). Genome completeness and contamination were checked using CheckM (52), and the final sequence was annotated using Prokka *v* 1.23 (53). The complete genome sequence was submitted to the NCBI under assembly accession number GCA_046097545.1 (BioProject number PRJNA835507, BioSample number SAMN45068613).

Multi-locus sequence typing (MLST) was performed using the *mlst* tool (https://github.com/tseemann/mlst) with the Institut Pasteur scheme (available at https://pubmlst.org/organisms/acinetobacter-baumannii). Resistance determinants were identified using AMRFinderPlus *v* 4.0 (54), and PHASTEST (55) or PHASTER (56) was used to detect prophage regions. The location of KL58 and OCL1 was identified using *Kaptive v 3* (57), with the most recent versions of the respective KL (12) and OCL (58) reference databases available at https://github.com/klebgenomics/Kaptive. *Prokka* v. 1.14.5 (53) output was used to extract coding sequences (*n* = 3,897) in faa file format, which were then screened for HMMs using *hmmscan,* available as part of the HMMER package *v 2.41.2* (59). The number of transmembrane segments per coding sequence was predicted using DeepTMHMM *v* 1.0 (60). Homologs of known or predicted function were identified using BLASTp (https://blast.ncbi.nlm.nih.gov/Blast.cgi).

### Genomic and temporal phylogenetic analysis of HTD outbreak isolates

Whole genome sequence alignment of BAL114 and BAL062 was constructed using pygenomeviz (https://github.com/moshi4/pyGenomeViz) with the BLAST CLI workflow. Short-read data in the NCBI SRA database available for the previously reported HTD outbreak isolates (26) were screened for *wzy1_Ph_* and *wzy2_Ph_* using BLASTn. Depolymerase genes were predicted using WebDePP (https://timskvortsov.github.io/WebDePP/) and HHPRED (https://toolkit.tuebingen.mpg.de/tools/hhpred) using the PHROGs *v4* database (61).

A core-SNP maximum likelihood phylogeny of CC2 lineage 2 HTD outbreak isolates, as well as two lineage 1 isolates representing the outgroup (Table S5), was constructed using Bactmap *v* 1.0.0 (https://github.com/nf-core/bactmap). Short-read data were mapped to the BAL062 reference genome (NCBI accession number LT594095.1), and regions of recombination were removed using Gubbins. A time-calibrated phylogeny using the Gubbins recombination-corrected tree was constructed using BactDating *v1.1.4* (https://github.com/xavierdidelot/BactDating). Four substitution models (strict gamma, relaxed gamma, mixed gamma*,* and *cARC*) were tested using 10^6^ iterations for Markov chain Monte Carlo (MCMC) settings. Following burn-in removal (10% of samples), convergence was tested to ensure effective sampling sizes were >100. Deviance information criterion (DIC) values for each model were then compared, revealing that the optimal model was continuous additive relaxed clock model (cARC) (62). The constructed tree was extracted and annotated in Adobe Illustrator.

### Global distribution analysis

BLASTp was used to screen coding sequences from publicly available genomes in the NCBI non-redundant and WGS databases (*n* = 25,742 as of 15 May 2025) for Wzy1_Ph_ and Wzy2_Ph_ using an aa identity and coverage cutoffs of 85%. Sequence, including ~10–40 kbp either side of the *wzy* gene identified in each genome, was aligned with the A320 CC2 reference genome (NCBI accession number CP032055.1) using BLASTn (https://blast.ncbi.nlm.nih.gov/Blast.cgi) to identify prophage boundaries and the location of insertion. Prophage sequences were then extracted and submitted to Phagescope (63) for taxonomic classification, calculation of completeness, and ORF annotation. Genome metadata was acquired from BioSample records using the NCBI data sets (64). KL and ST were identified as described above. RStudio *v. 3.3.0* + with ggplot2 (65) and/or tidyverse (66) packages were used to visualize distribution analyses. Construction of the bubble scatterplot included the additional geom_point function with the position_jitter option.

## SUPPLEMENTAL MATERIAL

Supplemental figures and tables spectrum.04084-25-s0001.pdf Figures S1 to S3 and Tables S1 to S5.

ASM does not own the copyrights to Supplemental Material that may be linked to, or accessed through, an article. The authors have granted ASM a non-exclusive, world-wide license to publish the Supplemental Material files. Please contact the corresponding author directly for reuse.
