# Supplementary material for: Multiple prophage acquisition events over the course of an outbreak drive lysogenic conversion of capsular polysaccharides produced by carbapenem-resistant Acinetobacter baumannii isolates
Source: Microbiol Spectr. 2026 Apr 27;14(6):e04084-25. doi: 10.1128/spectrum.04084-25 (PMC13228068; doi:10.1128/spectrum.04084-25)
Supplement: Supplemental figures and tables — Figures S1 to S3 and Tables S1 to S5. [file spectrum.04084-25-s0001.pdf]

Multiple prophage acquisition events over the course of an outbreak drive lysogenic conversion of capsular polysaccharides produced by carbapenem-resistant *Acinetobacter baumannii* isolates

Nowshin S. Sharar,[1] Christopher Harmer[2,3], Ruth M. Hall[2,3,†], Johanna J. Kenyon[4,5†,*]

[1] Centre for Immunology and Infection Control, School of Biomedical Sciences, Faculty of Health, Queensland University of Technology, Brisbane, Australia

[2] School of Life and Environmental Science, The University of Sydney, Sydney, Australia

[3] Sydney Infectious Diseases Institute, The University of Sydney, Sydney, Australia

[4] School of Pharmacy and Medical Sciences, Health Group, Griffith University, Parklands Drive, Gold Coast, Queensland, Australia

[5] Institute for Biomedicine and Glycomics, Griffith University, Parklands Drive, Gold Coast, Queensland, Australia.

† contributed equally

[*] To whom correspondence should be addressed: j.kenyon@griffith.edu.au

Table of Contents

SUPPLEMENTARY FIGURES

Supplementary Figure 1. Whole chromosome alignment of BAL114 and BAL062.

Supplementary Figure 2. Comparison of prophage regions carrying *wzy2*$_{Ph}$.

Supplementary Figure 3. Comparison of prophage regions carrying *wzy1*$_{Ph}$.

SUPPLEMENTARY TABLES

Supplementary Table 1. Distribution of *wzy1*$_{Ph}$ in *Acinetobacter* genome sequences.

Supplementary Table 2. Distribution of *wzy2*$_{Ph}$ in *Acinetobacter* genome sequences.

Supplementary Table 3. Prophage carrying *wzy2*$_{Ph}$ in *A. baumannii* genomes

Supplementary Table 4. Prophage carrying *wzy1*$_{Ph}$ in *A. baumannii* genomes

Supplementary Table 5. Accessions of isolates used in the phylogenetics analysis

SUPPLEMENTARY REFERENCES

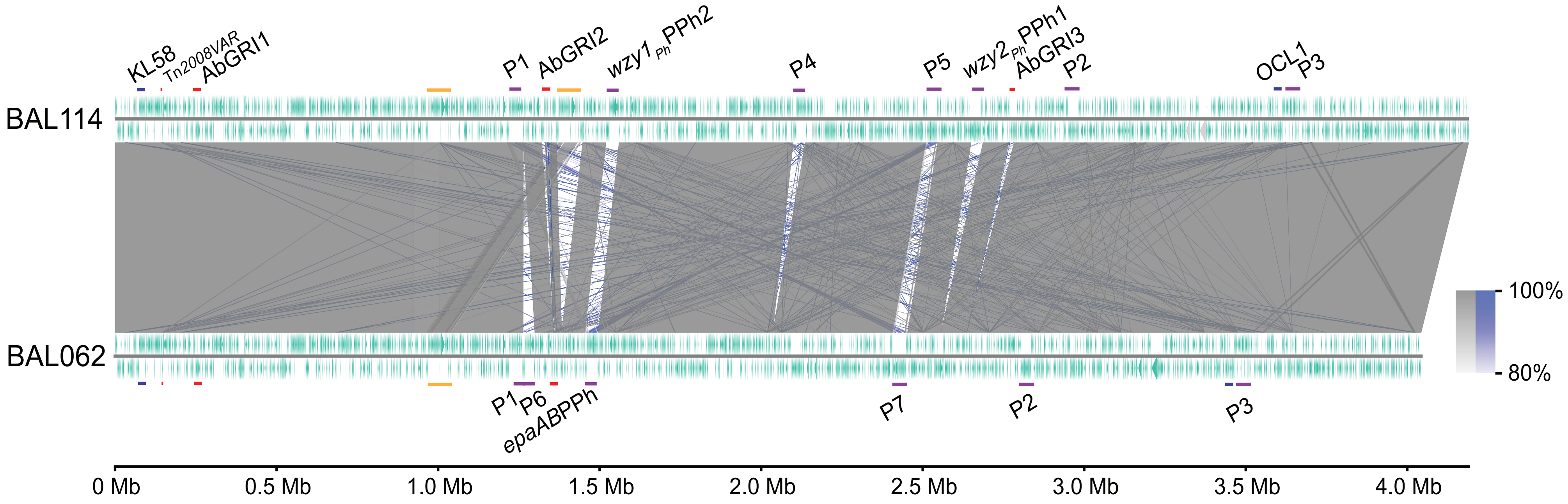

Supplementary Figure 1. Whole chromosome alignment of BAL114 with BAL062. Chromosome alignment. Polysaccharide loci, prophage and antibiotic resistance regions are indicated above. Grey and blue shading indicates regions of identity with blue denoting inversions. Shading key is shown to the right, and scale bar is shown below. Sequence has been reverse complemented to reflect the same chromosomal orientation used routinely. Figure generated with pygenomeviz (https://github.com/moshi4/pyGenomeViz) and annotated in Adobe Illustrator.

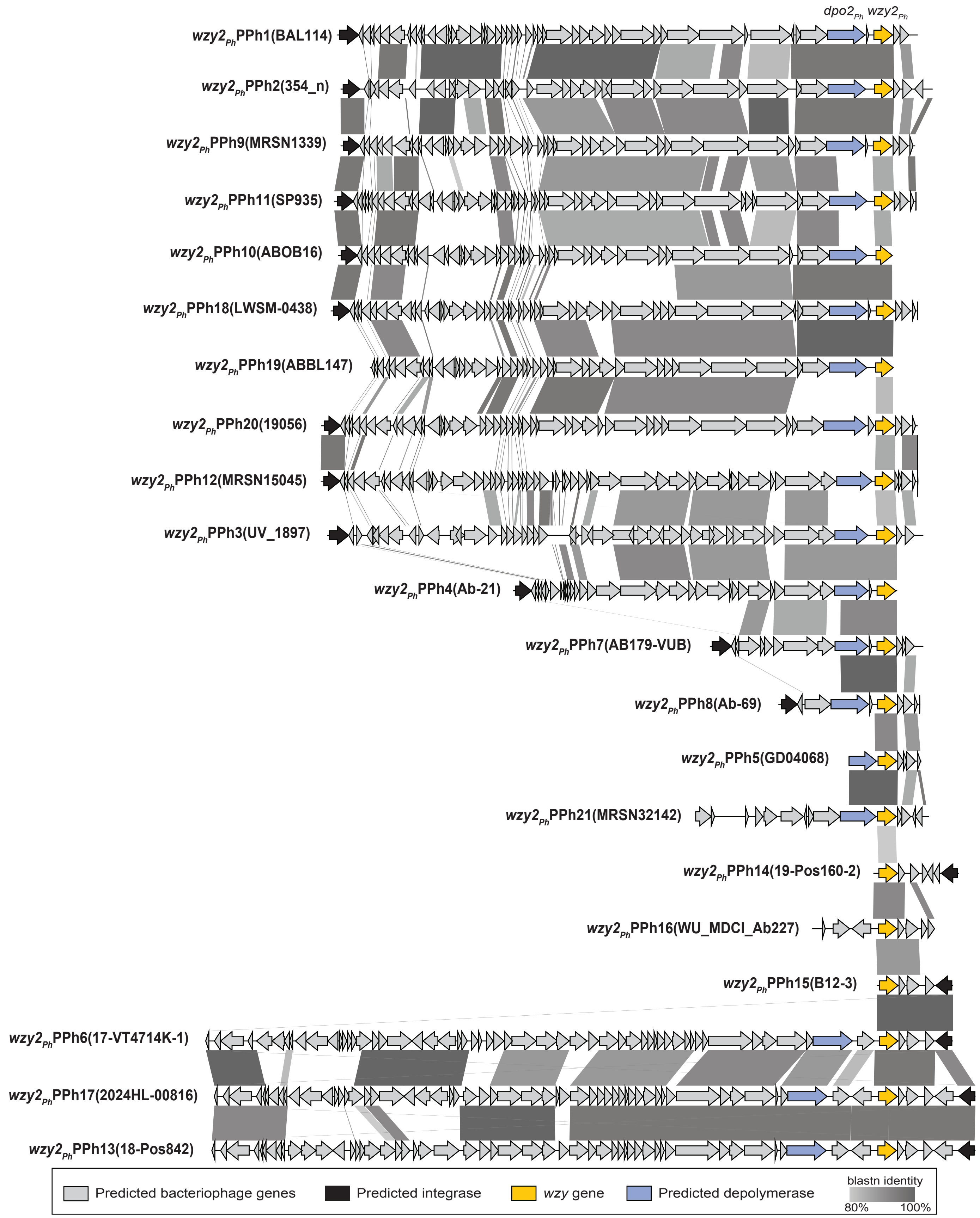

Supplementary Figure 2. Comparison of prophage regions carrying *wzy2$_{Ph}$*. Open reading frames are depicted as arrows coloured by scheme shown below. Figure drawn to scale using EasyFig (1) with grey shading indicating DNA sequence identity as shown by scale below.

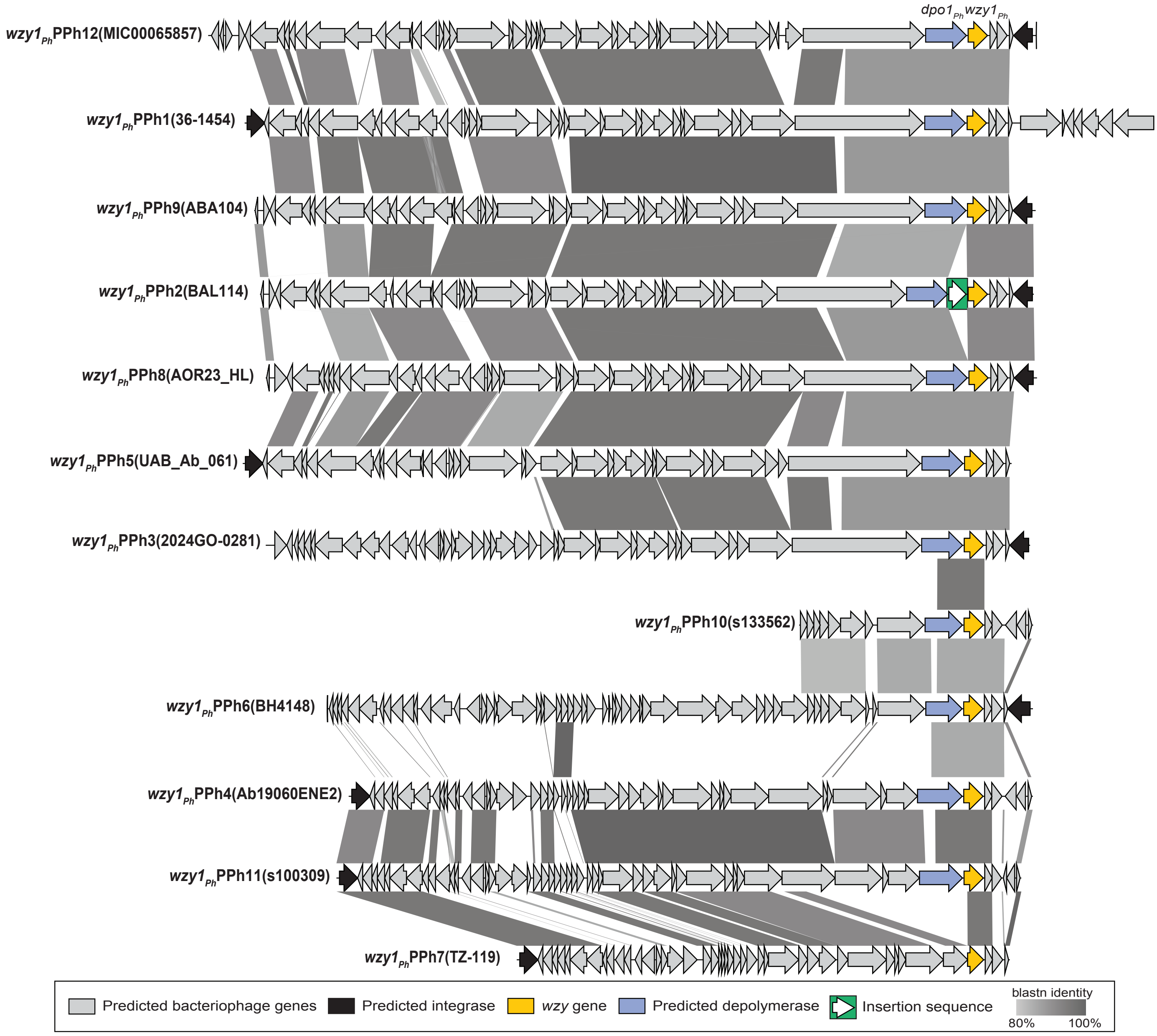

Supplementary Figure 3. Comparison of prophage regions carrying *wzy1*$_{Ph}$. Open reading frames are depicted as arrows coloured by scheme shown below. Figure drawn to scale using EasyFig (1) with grey shading indicating DNA sequence identity as shown by scale below.

Table S1. Distribution of *wzy1$_{Ph}$* in *Acinetobacter* genome sequences. HTD isolates are highlighted in blue, and other *Acinetobacter sp.* are highlighted grey.

Isolate name	NCBI accession number	Country	Year	Source[1]	ST	KL	Wzy$_{Ph1}$ accession	% ID (coverage)	Species	Prophage[2]
BAL114	GCA_046097545.1	Vietnam	2009	VAP	2	KL58	XLO26690.1	100 (100)	*A. baumannii*	*wzy1$_{Ph}$*PPh2
BAL_084	ERR190418	Vietnam	2009	VAP	2	KL58	-	100 (100)	*A. baumannii*	*wzy1$_{Ph}$*PPh2
36-1454	GCA_019192905.1	Russia	2013	Clinical	448	KL127	MBV6766733.1	99.14 (100)	*A. baumannii*	*wzy1$_{Ph}$*PPh1
2024GO-0281	GCA_040746295.1	USA	2024	HAI	25	KL127	ENK3501989.1	98.56 (100)	*A. baumannii*	*wzy1$_{Ph}$*PPh3
2024GO-0280	GCA_040746455.1	USA	2024	HAI	25	KL127	ENK3519221.1	98.56 (100)	*A. baumannii*	*wzy1$_{Ph}$*PPh3
2024GO-0288	GCA_041016385.1	USA	2024	HAI	25	KL127	ENM5942908.1	98.56 (100)	*A. baumannii*	*wzy1$_{Ph}$*PPh3
2024GO-0334	GCA_041297235.1	USA	2024	HAI	25	KL127	ENO7366806.1	98.56 (100)	*A. baumannii*	*wzy1$_{Ph}$*PPh3
2023EP-00156	GCA_031104545.2	USA	2023	Tissue	25	KL127	ELA7628119.1	98.56 (100)	*A. baumannii*	*wzy1$_{Ph}$*PPh3
Ab 19060 ENE2	GCA_042928675.1	Romania	2019	Environmental	33	KL127	HFE9913065.1	98.56 (100)	*A. baumannii*	*wzy1$_{Ph}$*PPh4
UAB_Ab_061	GCA_040445545.1	USA	2022	Blood	848	KL127	MES5709867.1	99.14 (100)	*A. baumannii*	*wzy1$_{Ph}$*PPh5
BH4148	GCA_030056085.1	Nigeria	2019	Blood	1137	KL127	MDI9751223.1	93.08 (100)	*A. baumannii*	*wzy1$_{Ph}$*PPh6
TZ-119	GCA_045050855.1	China	2020	Sputum	2034	KL127	MFK6097784.1	97.92 (83)	*A. baumannii*	*wzy1$_{Ph}$*PPh7
HX_S17	GCA_030364095.1	China	-	Blood	2034	KL127	WP_289344728.1	98.85 (100)	*A. baumannii*	*wzy1$_{Ph}$*PPh7
AOR23_HL	GCA_027855045.1	China	2020	Oral swab	2236	KL127	MDA3537749.1	95.68 (100)	*A. baumannii*	*wzy1$_{Ph}$*PPh8
ABA104	GCA_044264315.1	Singapore	2023	Blood	193	KL127	HFX6308378.1	97.98 (100)	*A. baumannii*	*wzy1$_{Ph}$*PPh9
s133562	GCA_049005065.1	China	2020	Hospital	459	KL127	MFX7353615.1	98.85 (100)	*A. baumannii*	*wzy1$_{Ph}$*PPh10
s100309	GCA_049003695.1	China	2016	Hospital	33	KL127	MFX5523801.1	99.14 (100)	*A. baumannii*	*wzy1$_{Ph}$*PPh11
MIC00065857	GCA_048960875	Bangladesh	2019	Blood	152	KL127	MFX2143529.1	98.85 (100)	*A. baumannii*	*wzy1$_{Ph}$*PPh12
1303.209	GCA_030140465.1	China	2021	-	-	-	WP_284077099.1	98.56 (100)	*A. nosocomialis*	-
1704.158	GCA_030177255.1	China	2021	-	-	-	WP_284077099.1	98.56 (100)	*A. nosocomialis*	-
AC1631	GCA_018139385.1	Malaysia	2016	Blood	-	-	MBR7749371.1	98.85 (100)	*A. nosocomialis*	-

[1] VAP = Ventillator associated pneumonia; HAI = Hospital acquired infection

[2] Region not determined for isolates of other species

Table S2. Distribution of *wzy2$_{Ph}$* in *Acinetobacter* genome sequences. HTD isolates are highlighted in blue, and other *Acinetobacter sp.* are highlighted grey.

Isolate name	NCBI accession no.	Country	Year	Source[1]	ST	KL	Wzy2$_{Ph}$ accession	% ID (cov)	Species	Prophage[2]
BAL114	GCA_046097545.1	Vietnam	2009	VAP	2	KL58	XLO29423.1	100 (100)	*A. baumannii*	*wzy2$_{Ph}$*PPh1
BAL_084	ERR190418	Vietnam	2009	VAP	2	KL58	-	-	*A. baumannii*	*wzy2$_{Ph}$*PPh1
354_n	ERR263728	Vietnam	2006	Carriage	2	KL58	-	-	*A. baumannii*	*wzy2$_{Ph}$*PPh2
277_ax	ERR197637	Vietnam	2005	Carriage	575	KL58	-	-	*A. baumannii*	*wzy2$_{Ph}$*PPh2
350_n	ERR263727	Vietnam	2006	Carriage	575	KL58	-	-	*A. baumannii*	*wzy2$_{Ph}$*PPh2
344_an	ERR263726	Vietnam	2006	Carriage	575	KL58	-	-	*A. baumannii*	*wzy2$_{Ph}$*PPh2
341_c	ERR263725	Vietnam	2006	Carriage	575	KL58	-	-	*A. baumannii*	*wzy2$_{Ph}$*PPh2
316_an	ERR263722	Vietnam	2006	Carriage	575	KL58	-	-	*A. baumannii*	*wzy2$_{Ph}$*PPh2
SIMBA005	GCA_047301355.1	Singapore	2006	Clinical	575	KL58	MFS1998311.1	100 (100)	*A. baumannii*	*wzy2$_{Ph}$*PPh2
UV_1897	ERR197570	Vietnam	2007	Tracheal wash	2	KL58	-	-	*A. baumannii*	*wzy2$_{Ph}$*PPh3
BAL_128	ERR190425	Vietnam	2010	VAP	2	KL58	-	-	*A. baumannii*	*wzy2$_{Ph}$*PPh3
BAL_339	ERR190488	Vietnam	2011	VAP	2	KL58	-	-	*A. baumannii*	*wzy2$_{Ph}$*PPh3
BAL_341	ERR190490	Vietnam	2011	VAP	2	KL58	-	-	*A. baumannii*	*wzy2$_{Ph}$*PPh3
BAL_346	ERR190491	Vietnam	2011	VAP	2	KL58	-	-	*A. baumannii*	*wzy2$_{Ph}$*PPh3
BAL_383	ERR190501	Vietnam	2012	VAP	2	KL58	-	-	*A. baumannii*	*wzy2$_{Ph}$*PPh3
BAL_369	ERR190498	Vietnam	2012	VAP	2	KL58	-	-	*A. baumannii*	*wzy2$_{Ph}$*PPh3
BAL_372	ERR190499	Vietnam	2012	VAP	2	KL58	-	-	*A. baumannii*	*wzy2$_{Ph}$*PPh3
BAL_377	ERR190500	Vietnam	2012	VAP	2	KL58	-	-	*A. baumannii*	*wzy2$_{Ph}$*PPh3
NCGM 193	GCA_016503145.1	Vietnam	2011	Clinical	571	KL58	HAV4825476.1	100 (100)	*A. baumannii*	*wzy2$_{Ph}$*PPh3
Ab-21	GCA_027088725.1	China	2019	Respiratory	490	KL170	MCZ3293617	96.94 (100)	*A. baumannii*	*wzy2$_{Ph}$*PPh4
GD04068	GCA_029836615.1	USA	2018	Surface swab	150	KL225	MDG9778644.1	93.88 (100)	*A. baumannii*	*wzy2$_{Ph}$*PPh5
GD03891	GCA_029840315.1	USA	2018	Surface swab	150	KL225	MDH0780837.1	93.88 (100)	*A. baumannii*	*wzy2$_{Ph}$*PPh5
17-VT4714K-1	GCA_028443225.1	Poland	2017	*Ciconia ciconia*	1019	KL41	MDC4992544.1	88.46 (99)	*A. baumannii*	*wzy2$_{Ph}$*PPh6
AB179-VUB	GCA_022459075.1	Belgium	2017	Sputum	10	KL58	UMN44917.1	100 (100)	*A. baumannii*	*wzy2$_{Ph}$*PPh7
Ab-69	GCA_027086195.1	China	2020	Urine	10	KL58	MCZ3090388.1	100 (100)	*A. baumannii*	*wzy2$_{Ph}$*PPh8
MRSN1339	GCA_016538425.2	USA	2010	Surveillance	154	KL58	EIB6893129.1	100 (100)	*A. baumannii*	*wzy2$_{Ph}$*PPh9
ABOB16	GCA_001416495.1	USA		Clinical	154	KL58	WP_154101149.1	100 (88)	*A. baumannii*	*wzy2$_{Ph}$*PPh10
SP935	GCA_010500415.1	India	2019	ETA	976	KL79	NDW25439.1	89.54 (100)	*A. baumannii*	*wzy2$_{Ph}$*PPh11
MRSN15045	GCA_016536535.2	USA	2003	Clinical	976	KL79	EHU3263679.1	90.56 (100)	*A. baumannii*	*wzy2$_{Ph}$*PPh12
18-Pos842	GCA_028441275.1	Poland	2018	*Ciconia ciconia*	1019	KL79	MDC4628787.1	88.46 (99)	*A. baumannii*	*wzy2$_{Ph}$*PPh13
19-Pos160-2	GCA_028440815.1	Poland	2019	*Ciconia ciconia*	2382	KL79	MDC4552419.1	88.72 (99)	*A. baumannii*	*wzy2$_{Ph}$*PPh14
B12-3	GCA_045985245.1	Nigeria	2024	Soil	2815	KL79	MFL9574829.1	88.46 (99)	*A. baumannii*	*wzy2$_{Ph}$*PPh15
WU_MDCI_Ab227	GCA_025407125.1	USA	2018	Connective tissue	123	KL79	MCT9459456.1	88.21 (99)	*A. baumannii*	*wzy2$_{Ph}$*PPh16

2024HL-00816	GCA_041271695.1	USA	2024	Rectal swab	150	KL94	ENO2604749.1	95.32 (98)	*A. baumannii*	*wzy2*$_{Ph}$PPh17
PUMA0150	GCA_033102935.1	Singapore	2023	Environmental	44	KL79	MDV7600345.1	88.21 (99)	*A. baumannii*	*wzy2*$_{Ph}$PPh17
LWSM-0438	GCA_034578615.1	Germany	2019/2020	Pig production settings	150	KL94	HEO1807084.1	94.96 (96)	*A. baumannii*	*wzy2*$_{Ph}$PPh18
UAB_Ab_159	GCA_040445205.1	USA	2022	BAL	150	KL94	MES5579095.1	95.32 (98)	*A. baumannii*	*wzy2*$_{Ph}$PPh18
UAB_Ab159	GCA_043038025.1	USA	2022	BAL	150	KL94	HFG6946200.1	95.32 (98)	*A. baumannii*	*wzy2*$_{Ph}$PPh18
ABBL147	GCA_001416315.1	USA	2012	Blood	150	KL94	WP_079745744.1	96.75 (94)	*A. baumannii*	*wzy2*$_{Ph}$PPh19
19056	GCA_050312035.1	Malaysia	2017	URT	2522	KL170	MGE9778986.1	89.8 (100)	*A. baumannii*	*wzy2*$_{Ph}$PPh20
MRSN32142	GCA_006492215.1	Germany	2006	Wound	155	KL41	TPT19382.1	93.88 (100)	*A. baumannii*	*wzy2*$_{Ph}$PPh21
Naval-57	GCA_000335535.1	USA	2006	Wound	155	KL41	ELX05999.1	93.88 (100)	*A. baumannii*	*wzy2*$_{Ph}$PPh21
FDAARGOS 1398	GCA_019046965.1	Germany	-		-	-	QWZ59887.1	88.72 (99)	*A. pittii*	-
LX-4	GCA_034296095.1	China	2019	Soil	-	-	MDY7373278.1	88.46 (99)	*A. oleivorans*	-
TZ-18	GCA_039887285.1	China	2023	Emergency Room	-	-	MEO4182981	88.97 (99)	*A. pittii*	-
1542444	GCA_000581835.1	USA	-	Perirectal	-	-	EXE61985.1	88.97 (99)	*Acinetobacter sp.*	-
TE2	GCA_000817365.1	India	2012	Surface of healthy skin	-	-	KIE87484.1	88.72 (99)	*A. pittii*	-
ULE_I039	GCA_048552035.1	Spain	2022	Pork meat	-	-	MFW1923702.1	89.29 (100)	*A. geminorum*	-

[1] VAP = Ventillator associated pneumonia; URT = Upper respiratory tract; ETA = Endotracheal Aspirate; BAL = Bronchioalveolar lavage.

[2] Region not determined for isolates of other species.

Table S3. Prophage carrying *wzy2$_{Ph}$* in *A. baumannii* genomes

Prophage	Size (bp)	Taxonomic assignment[1]	Completeness score[1]	Position of insertion[2]	Duplication length (bp)	% aa (% cov) Wzy2$_{Ph}$ to BAL114
wzy2$_{Ph}$PPh1(BAL114)	36,800	*Caudoviricetes*	100 (Medium)	1,550,165 – 1,550,150	16	-
wzy2$_{Ph}$PPh2(354_n)	37,543	*Caudoviricetes*	100 (Medium)	1,411,248 – 1,411,236	12	100 (100)
wzy2$_{Ph}$PPh3(UV_1897)	37,789	*Caudoviricetes*	100 (High)	1,550,165 – 1,550,150	16	100 (100)
wzy2$_{Ph}$PPh4(Ab-21)	24,351	*Caudoviricetes*	68.57 (High)	1,355,260 –	-	96.94 (100)
wzy2$_{Ph}$PPh5(GD04068)	4,587	*Caudoviricetes*	10.33 (Medium)	– 1,355,233	-	93.88 (100)
wzy2$_{Ph}$PPh6(17-VT4714K-1)	47,390	*Caudoviricetes*	100 (High)	2,599,790 – 2,599,784	7	88.46 (100)
wzy2$_{Ph}$PPh7(AB179-VUB)	13,536	*Caudoviricetes*	34.27 (Medium)	1,550,165 – 1,550,150	16	100 (100)
wzy2$_{Ph}$PPh8(Ab-69)	8,952	*Caudoviricetes*	17.23 (Medium)	1,411,248 – 1,411,236	12	100 (100)
wzy2$_{Ph}$PPh9(MRSN1339)	36,437	*Caudoviricetes*	100 (Medium)	2,000,654 – 2,000,645	10	100 (100)
wzy2$_{Ph}$PPh10(ABOB16)	35,134	*Caudoviricetes*	99.18 (Medium)	– 2,000,645	-	100 (88)
wzy2$_{Ph}$PPh11(SP935)	37,850	*Caudoviricetes*	100 (High)	1,411,248 – 1,411,236	12	89.54 (100)
wzy2$_{Ph}$PPh12(MRSN15045)	36,877	*Caudoviricetes*	100 (Medium)	1,411,248 – 1,411,236	12	90.56 (100)
wzy2$_{Ph}$PPh13(18-Pos842)	48,421	*Caudoviricetes*	100 (High)	2,599,790 – 2,599,784	7	88.46 (99)
wzy2$_{Ph}$PPh14(19-Pos160-2)	5,407	*Caudoviricetes*	11.11 (High)	– 2,599,784	-	88.72 (99)
wzy2$_{Ph}$PPh15(B12-3)	4,818	*Caudoviricetes*	9.89 (High)	– 2,599,784	-	88.46 (99)
wzy2$_{Ph}$PPh16(WU_MDCI_Ab227)	7,750	*Caudoviricetes*	15.94 (Low)	2,602,371 – 2,602,307	65	88.21 (99)
wzy2$_{Ph}$PPh17(2024HL-00816)	48,257	*Caudoviricetes*	100 (High)	2,599,784 – 2,599,784	1	88.21 (99)
wzy2$_{Ph}$PPh18(LWSM-0438)	37,223	*Caudoviricetes*	100 (High)	1,411,248 – 1,411,236	12	94.96-95.32 (96-98)
wzy2$_{Ph}$PPh19(ABBL147)	33,187	*Caudoviricetes*	93.68 (High)	– 3,207,251	-	96.75 (94)
wzy2$_{Ph}$PPh20(19056)	37,826	*Caudoviricetes*	100 (High)	-	-	89.8 (100)
wzy2$_{Ph}$PPh21(MRSN32142)	14,795	*Caudoviricetes*	37.29 (High)	– 1,411,236	-	93.88 (100)

[1] Determined by PhageScope

[2] Base positions of duplication in A320 genome determined via alignment with the first genome in which the prophage was found

Table S4. Prophage carrying *wzy1$_{Ph}$* in *A. baumannii* genomes

Prophage	Size (bp)	Taxonomic assignment[1]	Completeness score (confidence)[1]	Position of insertion[2]	Duplication length (bp)	% aa (% cov) Wzy1$_{Ph}$ to BAL114
wzy1$_{Ph}$PPh2(BAL114)	41,999	*Caudoviricetes*	100 (High)	2,599,787 – 2,599,784	4	-
wzy1$_{Ph}$PPh1(36-1454)	43,766	*Caudoviricetes*	100 (High)	2,188,518 – 2,188,494	25	99.14 (100)
wzy1$_{Ph}$PPh3(2024GO-0281)	41,486	*Caudoviricetes*	99.58 (High)	2,599,787 – 2,599,784	4	98.56 (100)
wzy1$_{Ph}$PPh4(Ab19060ENE2)	37,135	*Caudoviricetes*	100 (High)	1,355,260 – 1,355,233	28	98.56 (100)
wzy1$_{Ph}$PPh5(UAB_Ab_061)	41,731	*Caudoviricetes*	100 (High)	2,275,517 – 2,275,497	21	99.14 (100)
wzy1$_{Ph}$PPh6(BH4148)	38,304	*Caudoviricetes*	100 (Medium)	899,931 – 899,914	18	93.08 (100)
wzy1$_{Ph}$PPh7(TZ-119)	26,758	*Caudoviricetes*	74.37 (Medium)	1,355,260 – 1,355,230	31	97.92 (83)
wzy1$_{Ph}$PPh8(AOR23_HL)	41,862	*Caudoviricetes*	99.25 (High)	2,599,784 – 2,599,674	111	95.68 (100)
wzy1$_{Ph}$PPh9(ABA104)	42,412	*Caudoviricetes*	100 (High)	2,599,784 – 2,599,674	111	97.98 (100)
wzy1$_{Ph}$PPh10(s133562)	12,693	*Caudoviricetes*	33.36 (High)	1,355,233 –	-	98.85 (100)
wzy1$_{Ph}$PPh11(s100309)	37,173	*Caudoviricetes*	100 (Medium)	1,355,260 – 1,355,230	31	99.14 (100)
wzy1$_{Ph}$PPh12(MIC00065857)	44,948	*Caudoviricetes*	100 (Medium)	2,599,787 –	-	98.85 (100)

[1] Determined by PhageScope

[2] Base positions of duplication in A320 genome determined via alignment with the first genome in which the prophage was found.

Table S5. Accessions of isolates used in the phylogenetics analysis

Isolate	Accession	Year of isolation
BAL062	LT594095	2009
91_an	ERR197594	2004
295_an	ERR197643	2005
354_n	ERR263728	2006
BAL_114	SRR31521410	2009
BAL_058	ERR190415	2009
BAL_084	ERR190418	2009
BAL056	ERR190414	2009
BAL064	ERR190417	2009
UV_1897	ERR197570	2007
BAL128	ERR190425	2010
BAL215	ERR190448	2010
BAL219	ERR190450	2010
BAL315	ERR190478	2011
BAL295	ERR190473	2011
BAL298	ERR190474	2011
BAL225	ERR190452	2010
BAL230	ERR190454	2010
BAL238	ERR190457	2010
BAL341	ERR190490	2011
BAL346	ERR190491	2011
BAL350	ERR190493	2011
BAL369	ERR190498	2012
BAL372	ERR190499	2012
BAL377	ERR190500	2012
BAL383	ERR190501	2012
235_ax	ERR197624	2005
242_an	ERR197627	2005
UV_1261	ERR197568	2005
230_ax	ERR197622	2005
BAL_256	ERR190465	2011
BAL_291	ERR190472	2011
BAL_276	ERR190469	2011
BAL_306	ERR190475	2011
BAL_320	ERR190479	2011
BAL_321	ERR190480	2011
BAL_331	ERR190484	2011
BAL_194	ERR190438	2010
BAL_353	ERR190494	2011
BAL_339	ERR190488	2011
BAL_359	ERR190495	2011
BAL_361	ERR190496	2011
BAL_186	ERR190433	2010
BAL_188	ERR190434	2010
BAL_190	ERR190435	2010
BAL_191	ERR190436	2010
BAL_202	ERR190440	2010
BAL_182	ERR190432	2010
BAL_262	ERR190466	2011
BAL_173	ERR190430	2010
BAL_208	ERR190444	2010
BAL_235	ERR190456	2010
BAL_242	ERR190459	2011
BAL_244	ERR190461	2011
BAL_253	ERR190462	2011
BAL_224	ERR190451	2010

SUPPLEMENTARY REFERENCES

1. Sullivan MJ, Petty NK, Beatson SA. 2011. Easyfig: a genome comparison visualizer Bioinformatics 27:1009-1010.
